# Elevated temperatures have sex-specific effects on nuptial gift behavior

**DOI:** 10.1093/beheco/araf049

**Published:** 2025-05-08

**Authors:** Matilda Q R Pembury Smith, Laura Latkova, Rhonda R Snook

**Affiliations:** Department of Zoology, Stockholm University, Stockholm, SE-106 91, Sweden; Bolin Centre for Climate Research, Stockholm University, Stockholm, SE-106 91, Sweden; Department of Zoology, Stockholm University, Stockholm, SE-106 91, Sweden; Department of Zoology, Stockholm University, Stockholm, SE-106 91, Sweden; Bolin Centre for Climate Research, Stockholm University, Stockholm, SE-106 91, Sweden

**Keywords:** climate change, mate choice, nuptial gift, pre-copulatory sexual selection, temperature

## Abstract

Increasing developmental temperatures are well-known to impact fertility, yet their effects on pre-copulatory behaviors, despite having clear fitness consequences, are often overlooked. In many species, male nuptial gift presentation during courtship plays an important role in sex-specific mate choice, fitness and subsequent co-evolutionary dynamics. However, developmental temperature effects on nuptial gift behaviors and their implications for population fitness remain unknown. Heat-induced changes to male behavior may signal fertility, diving female discrimination, particularly in monandrous systems where exclusively pairing with an infertile male threatens population growth. Additionally, as nuptial gift production is costly, the differential allocation hypothesis suggests males should adjust gift investment based on female fitness. Here, we investigated how elevated developmental temperature affects nuptial gift behavior, mating likelihood and reproductive output in the monandrous species *Drosophila subobscura*. Individuals developed at either a control or stressful temperature, and fully factorial no-choice mating tests were used to identify sex-specific effects of heat stress. Heat-stressed males were largely infertile, less likely to mate, present a gift, or have a gift accepted, suggesting nuptial gifts may signal male fertility and influence female mate choice. Heat-stressed females were also less likely to mate or receive a gift, and were presented with fewer gifts from heat-stressed males. As heat-stressed females required more gifts to match the reproductive output of controls, selection may drive male mate choice through strategic resource allocation. These findings highlight how climate change may significantly impact sex-specific mate choice, with important implications for selection on pre-copulatory courtship traits and population dynamics.

## Teaser

Abiotic conditions may alter nuptial gift-giving and acceptance, impacting sex-specific fitness. Using the monandrous fruit fly (Drosophila subobscura), we tested how developmental temperature affects nuptial gift behavior and fitness in both sexes. Heat-stressed males were infertile, less likely to present a gift or have a gift accepted, but strategically allocated gifts based on female heat treatment. Heat-stressed females required more gifts to maintain fecundity. Results demonstrate mutual mate choice with important implications for population growth as temperatures rise.

## Introduction

Global temperatures and instances of prolonged heatwaves are rising (Calvin et al. 2023). Ectotherms may be particularly affected since physiological performance is tightly constrained by external temperatures (Huey and Stevenson 1979; Parmesan 2006). Ectotherm thermal tolerance is typically inferred using Critical Thermal Limits (CTL: CT_max_/CT_min_; Angilletta 2009), the temperature boundaries beyond which biological functions fail (eg Kellermann et al. 2009, 2012; Diamond et al. 2012; Hoffmann et al. 2013; Kingsolver et al. 2013). However, sublethal temperatures can compromise fertility and fecundity (Walsh et al. 2019a), with Thermal Fertility Limits (TFL)—the temperature boundaries beyond which an individual remains fertile (Bretman et al. 2024)—better reflecting both current species’ geographic distributions (Parratt et al. 2021; van Heerwaarden and Sgrò 2021) and extinction risk (van Heerwaarden and Sgrò 2021). Fertility responses to temperature can differ between the sexes. For example, female fruit flies (*Drosophila* spp.) tolerate temperatures 1.3 °C higher than males before reaching 50% sterility (van Heerwaarden and Sgrò 2021). Despite this, research examining sex-specific temperature effects on reproduction remain limited (Walsh et al. 2019b; Dougherty et al. 2024). Ignoring these differences can lead to inaccurate assessments of species’ responses to climate change.

In addition to fertility, temperature changes can alter pre-copulatory mating-related behavior, particularly if they elicit sex-specific behavioral responses (Ritchie et al. 2001; Candolin 2019; Rosenthal and Elias 2019, 2025; Leith et al. 2021). Acute heat stress during courtship can alter ectotherm activity levels and metabolism, whereas developmental exposure can induce lasting morphological, physiological and behavioral changes, shaping future courtship signals and resource allocation (García-Roa et al. 2020; Leith et al. 2021). Therefore, temperature shifts can substantially modulate behavioral interactions among mating partners by affecting both the attractiveness of the courting individual and the chooser’s behavior (eg Brandt et al. 2018; Macchiano et al. 2023). However, the effects of elevated adult and developmental temperature on mating behavior remain understudied compared to other ecologically important traits (Leith et al. 2021) such as acoustic signaling (Narins and Meenderink 2014), migration (Jonsson and Jonsson 2018) and prey capture (Kruse et al. 2008).

Developmental heat stress can result in adult male infertility because, in many ectothermic taxa, spermatogenesis begins at this stage (eg David et al. 2005; Sales et al. 2021; Walsh et al. 2021; Baur et al. 2021). Male mating signals, which demand substantial energy investment, are thought to reflect male fertility (Sheldon 1994) and/or quality (Andersson 1994), as they are often condition dependent. Therefore, changes to male courtship following developmental heat stress may reliably signal male fertility status, allowing females to adaptively avoid mating with sterile mates. Despite the significant implications for population viability and performance (Candolin and Heuschele 2008), the effects of temperature on pre-copulatory traits are often examined separately from fertility and fecundity, though recent studies are beginning to address this gap (Sutter et al. 2019; Vasudeva et al. 2021; Wai Mak et al. 2023). Examining how developmental temperature affects both mating behavior and reproductive output can improve our ability to predict population responses to climate warming.

Nuptial gifts—non-gametic genetic or nutritional material transferred during courtship or copulation—are an important aspect of pre-copulatory behavior in many taxa (Lewis and South 2012). For males, gift presentation can function as mating effort, increasing copulation likelihood, or paternal investment, increasing offspring production (Steele 1986a; Ivy et al. 1999; Engqvist 2007; Pärssinen et al. 2024 but see Vahed 2003; Rines et al. 2023) and survival (Simmons and Parker 1989; Vahed 1998; Cardoso and Gilbert 2007; Alexandre et al. 2011; Lewis and South 2012). By accepting a gift, females are therefore provided with direct fecundity benefits, in addition to nutritional supplementation which can increase female longevity (Wiklund et al. 1993; Arnqvist and Nilsson 2000; Voigt et al. 2008; Browne and Gwynne 2023). The direct benefits of gift acceptance to both sexes can increase with multiple nuptial gifts (Vahed 1998). For example, in *Drosophila subobscura*, both the mating success of the male (Immonen et al. 2009) and the nutritional advantage to the female (Steele 1986a; Immonen et al. 2009) increased as gift acceptance increased. However, nuptial gifts can also be costly to both sexes. Males invest significant energy in nuptial gift production (Voigt et al. 2005; Lewis et al. 2014; Liu et al. 2015), driving the evolution of false gifts to decrease mating costs (“Medea gifts” Arnqvist and Nilsson 2000; eg Preston-Mafham 1999; Sakaluk 2000; Albo et al. 2011). For females, males can exploit sensory biases, manipulating females into suboptimal matings (“sensory trap”; Christy 1995), a central hypothesis in the evolution and maintenance of nuptial gifts (Vahed 2007). For example, gift acceptance can slow female re-mating rates below an optimum (Simmons and Gwynne 1991; Sakaluk et al. 2006; Pärssinen et al. 2024), and decrease sperm transfer in subsequent matings (Rines et al. 2023). Thus, nuptial gifts influence the co-evolutionary dynamics between the sexes, via both sexual selection and sexual conflict (Arnqvist and Nilsson 2000; Vahed 2007; Gwynne 2008).

Abiotic conditions can alter nuptial gift presentation and acceptance, impacting sex-specific mate choice. Variation in nutritional resources, for example, can influence male gift giving behavior and the quality of the gifts themselves, signaling male nutrition status, which influences female pre-copulatory mate preferences (Wedell and Ritchie 2004; Bussière et al. 2005; Immonen et al 2009; Albo et al. 2011). Therefore, gift-giving is thought to represent an honest signal of male quality (in species that do not produce a false gift; Gwynne 2008; Albo et al. 2011). Additionally, given the energetic cost of gift production, sexual and natural selection are expected to drive male strategic investment in response to perceived female quality (Solano-Brenes et al. 2021), with lower quality (eg starved) males showing greater discrimination as they incur a greater cost (Engqvist and Sauer 2001, 2002). Thus, male strategic allocation of gifts can function as a form of male mate choice (Edward and Chapman 2011). However, most studies examining how abiotic conditions influence nuptial gift behavior have focused on nutrition (eg Wedell and Ritchie 2004; Engels and Sauer 2006; Immonen et al. 2009; Albo et al. 2011; Brown 2011; Macedo-Rego et al. 2016; Solano-Brenes et al. 2021; but see Fox et al. 2006; Walsh et al. 2017), with limited insight on how other factors, such as developmental temperature, impact nuptial gift behavior in both sexes, and how this influences sex-specific mate choice.

The fruit fly, *Drosophila subobscura*, distributed across Europe and the Americas, serves as a model organism for studying nuptial gift behavior due to the ease of observing both gift presentation and acceptance. During courtship, males transfer regurgitated crop content to females, which they accept with their proboscis (Steele 1986b). This exchange can occur multiple times before mating (Immonen et al. 2009), and provides a nutritious benefit to the female (Steele 1986a; Immonen et al. 2009). *Drosophila subobscura* has a monandrous mating system, with previous work observing low (3.4%-4%; Fisher et al. 2013) or no instances of female remating (Maynard Smith 1956; Holman and Snook 2006; Lizé et al. 2012). Therefore, nuptial gifts serve as both mating and paternal effort as greater male investment carries little risk of supporting another male’s offspring, but may limit future mating opportunities (Immonen et al. 2009). Additionally, *D. subobscura* provides an excellent model system for studying thermal effects on reproduction, as developmental heat stress has previously been shown to decrease fertility (Fisher et al. 2013; Santos et al. 2023) and sperm motility, negatively impacting progeny production (Porcelli et al. 2017).

In this study, we examined how developmental heat stress affects nuptial gift behavior and reproductive output in *D. subobscura*. Males and females were exposed throughout development to either a control 18 °C (Santos 2007; Castañeda et al. 2013) or a stressful but sublethal 25 °C temperature treatment that causes high male sterility (Moreteau et al. 1997). Following developmental temperature exposure, no-choice mating tests were conducted on eclosed adults, tested at the control temperature, to delineate sex-specific developmental temperature effects on nuptial gift behavior. While a recent study on this species investigated gift-giving in heat-stressed males (Grandela et al. 2024), female temperature was not examined, and only the likelihood of gift giving and acceptance was reported. Since temperature likely affects multiple aspects of gift behavior—such as the number and timing of gifts presented and accepted—and these effects can be sex-specific, influencing intersexual interactions and sexual conflict, our study offers a broader understanding of putative population consequences under a warming climate.

## Methods

### Stocks

In 2022 wild caught individuals (between coordinates 55°55’13.1“N 3°11’32.0”W and 55°55’56.3”N 3°11’42.4”W) from the United Kingdom were used to produce isofemale lines, and four were randomly selected (DG-7, BF1, -7, -14) due to the scale and reciprocal nature of the experiment. In the lab, all lines were housed at 18 °C in standard culture vials containing 5ml of a standard food medium (1L water: 80g medium cornmeal, 18g dried yeast, 10g soya flour, 80g malt extract, 40g molasses, 8g agar, 25 mL of 10% Nipagin, 4 mL of propionic acid) at 12-h light:12-h dark cycle. These stocks were used to generate experimental animals. No ethical approval was required for the work.

### Production of focal individuals

**Fig. 1.**
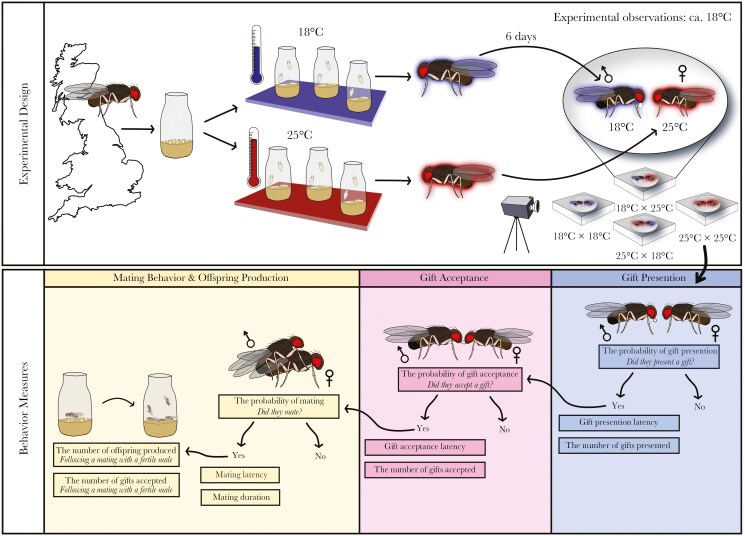
**A schematic of the experimental design and the behaviors measured and analyzed.** Wild caught individuals of *Drosophila subobscura* from the United Kingdom were allowed to lay eggs for 3 d or until ca. 30 eggs were present in each vial. Vials were then placed at either 18 °C or 25 °C throughout development until pupal eclosion. Once eclosed, virgin individuals of each sex were collected and kept at 18 °C. Six days after eclosion (to ensure sexual maturity), one male (18 °C or 25 °C) and one female (18 °C or 25 °C) from the same line were introduced into a 3D-printed black plastic chamber. This produced 4 possible pairing groups for each line: 1 control group (18 °C male x 18 °C female) and three treatment groups (25 °C male x 25 °C female; 18 °C male x 25 °C female and 25 °C male x 18 °C female). All pairs were recorded by a camcorder for 2 h. During this observation period we noted three gift presentation behaviors: (*i*) whether a gift was presented, (*ii*) gift presentation latency and (*iii*) the total number of gift presentations. Given a gift was presented, we also noted three gift acceptance behaviors: (*i*) whether a gift was accepted (*ii*) gift acceptance latency, and (*iii*) the total number of gifts accepted by the female. Gift observations were carried out until either copulation began (if a mating occurred), or for 120 min (if a mating did not occur). We also noted whether a mating occurred, mating latency and duration. These three variables were analyzed only if an individual accepted a gift. When a mating occurred, females were moved to a new vial and left to lay eggs for 30 d to measure long term reproductive output. For those mated to a fertile control male, we also examined the number of offspring produced. For details on all models and sample sizes see supplementary data.

To generate focal individuals, each line was placed in food vials. Each vial had a ca. 1:1 sex ratio and 20 individuals per vial. Parent flies were removed after three days or until ca. 30 eggs were present (to ensure approximately equal egg density) (Fig. 1). For all experiments, 18 °C was used as a control treatment (Santos 2007; Castañeda et al. 2013), and 25 °C as a thermally stressful treatment which is within this species thermal range (Moreteau et al. 1997) (Fig. 1). After the removal of the parental flies, vials from each line were kept at either of these two developmental temperatures (Fig. 1). Virgin focal offspring were collected within 24h after eclosion under light CO_2_ anesthesia, sexes were housed separately with either 10 females or 1 male per vial at 18 °C. Males were housed separately as high male density in this species can increase male-male aggression, which decreases the probability of mating (Lizé et al. 2014).

### Behavioral observations and trait measurements

Six days after eclosion, to ensure sexual maturity (Holman et al. 2008), one male (18 °C or 25 °C) and one female (18 °C or 25 °C) from the same line were introduced into a 3D-printed black plastic chamber, consisting of a cuboid of 34 mm x 33 mm x 9 mm with a hemispherical cavity of diameter 20 mm and depth 7 mm (Hopkins et al. 2019). All pairs were introduced within 30 min of the light period of our photoperiod beginning. This provides a “dawn” stimulus, when *D. subobscura* are most active in nature (Shorrocks and Charlesworth 1980). A no-choice mating design was used (Dougherty and Shuker 2015), producing 4 possible pairing groups for each line: 1 control group (18 °C male x 18 °C female, *n* = 104) and three treatment groups (25 °C male x 25 °C female, *n* = 77; 18 °C male x 25 °C female, *n* = 76 and 25 °C male x 18 °C female, *n* = 78) (Fig. 1; Table S3).

All pairs were recorded by a camcorder (Sony HDR-CX405 or Panasonic HC-V180) for two hours. During this observation period we noted (*i*) whether a gift was presented, (*ii*) gift presentation latency, (*iii*) the total number of gift presentations, (*iv*) if a gift was presented, whether it was accepted, (*v*) gift acceptance latency, and (*vi*) the total number of gifts accepted (Fig. 1). Observations were carried out until either copulation began (if a mating occurred), or for 120 min (if a mating did not occur). A 120-min observation period was chosen as most *D. subobscura* that mate within two hours do so within the first 60 min and, as this species is most active just after dawn, two hours is a reasonable estimate of this active interaction period (Fisher et al. 2013). Mating latency and copulation duration were also recorded if mating occurred (Fig. 1). After copulation, males were discarded and females were transferred into individual vials where they were left to lay eggs for 30 d to monitor long-term reproductive success. All behavioral observations were conducted over 23 d between 2023-03-23 and 2023-05-04 in a temperature-controlled room (ca. 18 °C; Fig. 1) with high-intensity light (900-1500 lx) which facilitates mating in this species (Wallace and Dobzhansky 1946). Video recordings were replayed and analyzed manually. Three observers analyzed the videos. To ensure consistency between analyzers, observers quantified traits of interest in a series of practice videos. Observers achieved 95% (± 0.03%) alignment across assessments for all traits before collecting data from the experimental videos. During video playback, each mating pair was described using an anonymous ID so video analyzers did not know the temperature pairing, reducing potential experimenter bias.

### Statistical analysis

All analyses were conducted in R v 4.2.2 (R Core Team 2022). The ‘glmmTMB’ package v 1.1.7 was used to generate statistical models and the ‘ggplot2’ package v 3.4.3 was used to generate all figures (Wickham 2016). The significance of main effects, interactions and covariates (if included) were evaluated using the *Anova()* function (type III) from the ‘car’ package v 3.1-2. Post hoc comparisons of significant main level effects and interactions were performed using the function *emmeans* and *emtrends* in the ‘emmeans’ package v 1.8.8. All analyses were done using linear models. Descriptions of each model and model outputs (Table S1), individual line sample sizes (Table S3) and summary statistics (Table S4) are found in the supplementary data file.

For all models (unless stated otherwise, see below), male temperature (18 °C and 25 °C), female temperature (18 °C and 25 °C) and their two-way interaction were included as fixed effects. Genetic line was also included as a fixed effect for all models. However, interactions between line and the variables of interest were not tested as *(i)* lines were randomly selected, and *(ii)* estimating individual line effects were not a primary focus as we aimed to make broader inferences on population behavior. However, line means are visually represented and estimates for the effect of line are outlined in Table S1.

Given the hierarchical nature of the behavioral traits measured, we used a structured analytical approach (see “Behavioral Measures” in Fig. 1). First, we examined the probability a gift was presented. For males that presented a gift, we then analyzed gift-presentation latency and the number of gifts presented. In the latter model, the time over which measurements were taken was included as a covariate as behavioral monitoring stopped when an individual mated, leading to variation in the time over which gifts were counted between pairs.

A small subset of females (*n* = 19) mated without accepting a gift, mostly from pairs that developed at 18 °C, who displayed abnormal mating behavior, engaging in mating latencies that were significantly longer or shorter than average (Levene’s Test: *F *= 6.050, *P* < 0.05). These individuals were therefore excluded from subsequent analysis.

Given a gift was presented, we next examined the probability a gift was accepted. For females that accepted a gift, we then analyzed gift-acceptance latency and the number of gifts accepted. Gift presentation latency was included as a covariate when analyzing gift acceptance latency to account for its influence on female gift acceptance time, independent of temperature. When analyzing the number of gifts accepted, the number of gifts presented and the time over which measurements were taken were included as covariates. We also examined the probability of mating and, for pairs that mated, mating latency and copulation duration.

Finally, as the 25 °C temperature treatment rendered males almost entirely sterile, subsequent analyses of offspring production were restricted to females mated to fertile control males. In this analysis, female temperature, the number of gifts accepted and line were included as fixed effects. An interaction between female temperature and gift acceptance was initially evaluated using log-likelihood analysis (Table S2), but was subsequently excluded. Additionally, we conducted a follow-up analysis to assess the effect of female temperature on the number of gifts accepted only in these mated individuals. Here, only female temperature and line were included as fixed effects.

## Results

### Male and female heat stress decreases the probability of male gift presentation

**Fig. 2.**
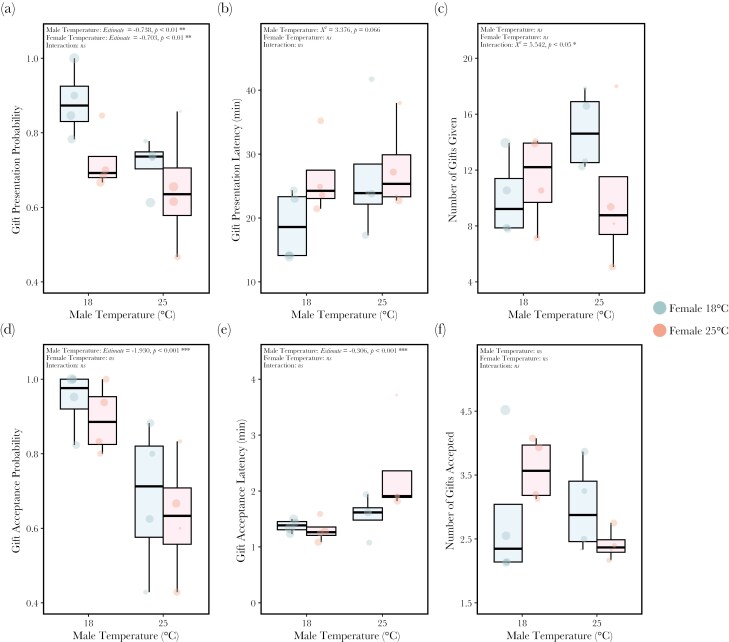
**The effect of male and female development temperature on (a) gift presentation probability, (b) gift presentation latency, (c) the number of gifts presented, (d) gift acceptance probability, (e) gift acceptance latency, and (f) the number of gifts accepted.** One male and one female from either treatment (female: 18 °C blue or 25 °C red) from the same line were introduced into a chamber. Gift presentation latency, the number of gifts presented and gift acceptance probability were examined in pairs in which the male presented a gift. Gift acceptance latency and the number of gifts accepted were examined in pairs in which the female accepted a gift. The recording of gift behavior stopped when a pair began copulating. If a pair did not copulate, behavior was monitored for 120 min. Each point represents the mean value for each isofemale line and the diameter of each point is representative of sample size (see Table S3).

Developmentally heat-stressed males (hereafter heat-stressed males) were 20% less likely to present a gift compared to control males (Fig. 2A; *Estimate* = -0.738 ± 0.27, *z* = -2.703, *P* < 0.01). Similarly, developmentally heat-stressed females (hereafter heat-stressed females) were 17% less likely to have a gift presented to them compared to control females (Fig. 2A; *Estimate* = -0.703 ± 0.27, *z* = -2.609, *P* < 0.01). There was no significant interaction between male and female temperature on the probability a male presented a gift (ANOVA: *X*^*2*^* *= 2.251, *df* = 1, *P* = 0.134).

### The effect of male temperature on the number of gifts presented depends on female developmental temperature

Given a gift was presented, there was no significant effect of male temperature (Fig. 2B; ANOVA: *X*^*2*^* *= 3.376, *df* = 1, *P* = 0.066), female temperature (ANOVA: *X*^*2*^* *= 1.650, *df* = 1, *P* = 0.199) or the interaction between male and female temperature (ANOVA: *X*^*2*^* *= 0.009, *df* = 1, *P* = 0.926) on the time it took for the male to present a gift.

However, there was a significant interaction between male and female temperature on the number of gifts males presented (Fig. 2C; ANOVA: *X*^*2*^* *= 5.542, *df* = 1, *P* < 0.05). Heat-stressed females received fewer gifts compared to control females, but only when the male was also heat stressed (Male 18 °C: *Estimate* = 0.011 ± 0.17, *z* = 0.065, *P* = 0.948; Male 25 °C: *Estimate* = -0.571 ± 0.19, *z* = -3.067, *P* < 0.01).

### Male heat stress decreases the probability that females accept a gift

Heat-stressed males were 36% less likely to have a gift accepted compared to control males (Fig. 2D; *Estimate* = -1.930 ± 0.42, *z* = -4.565, *P* < 0.001). Neither female temperature (ANOVA: *X*^*2*^* *= 1.244, *df* = 1, *P* = 0.265) nor the interaction between male and female temperature (ANOVA: *X*^*2*^* *= 0.091, *df* = 1, *P* = 0.763) had a significant effect.

### Male heat stress increases gift acceptance latency but the number of gifts accepted is not impacted by temperature after accounting for the number of gifts presented

Females were significantly slower to accept gifts from heat-stressed males compared to control males (Fig. 2E; *Estimate *= -0.306 ± 0.09, *t* = -3.529, *P* < 0.001). There was no significant effect of female temperature (ANOVA: *X*^*2*^* *= 1.581, *df* = 1, *P* = 0.209) or the interaction between male and female temperature (ANOVA: *X*^*2*^* *= 0.362, *df* = 1, *P* = 0.548).

Accounting for the number of gifts presented, the number of gifts accepted by females was not significantly affected by male temperature treatment (ANOVA: *X*^*2*^* *= 1.144, *df* = 1, *P* = 0.285), female temperature treatment (ANOVA: *X*^*2*^* *= 0.768, *df* = 1, *P* = 0.381) or their interaction (ANOVA: *X*^*2*^* *= 1.411, *df* = 1, *P* = 0.235) (Fig. 2F).

### Male and female heat stress reduces mating probability but does not affect mating latency or copulation duration

**Fig. 3.**
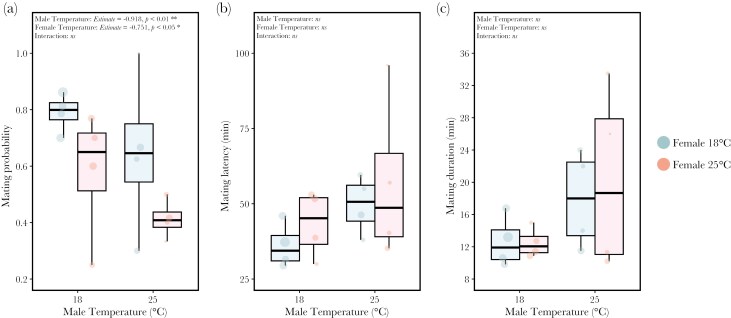
**The effect of male and female development temperature on (a) mating probability, (b) mating latency, and (c) mating duration.** One male and one female from either treatment (female: 18 °C blue or 25 °C red) from the same line were introduced into a chamber and mating behavior was monitored. Mating behavior was analyzed on pairs in which gift acceptance occurred. Each point represents the mean value for each isofemale line and the diameter of each point is representative of sample size (see Table S3).

Despite gift acceptance, male (*Estimate* = -0.918 ± 0.34, *z* = -2.683, *P* < 0.01) and female (*Estimate* = -0.751 ± 0.34, z = -2.219, *P* < 0.05) heat stress significantly reduced the probability of mating (Fig. 3A). There was no significant interaction between male and female temperature (ANOVA: *X*^*2*^* *= 0.326, *df* = 1, *P* = 0.568). Neither mating latency (Fig. 3B) nor copulation duration (Fig. 3C) were significantly influenced by male temperature (mating latency ANOVA: *X*^*2*^* *= 1.946, *df* = 1, *P* = 0.163; copulation duration ANOVA: *X*^*2*^* *= 3.254, *df* = 1, *P* = 0.071), female temperature (mating latency ANOVA: *X*^*2*^* *= 1.814, *df* = 1, *P* = 0.178; copulation duration ANOVA: *X*^*2*^* *= 0.096, *df* = 1, *P* = 0.757) or their interaction (mating latency ANOVA: *X*^*2*^* *= 0.197, *df* = 1, *P* = 0.657; copulation duration ANOVA: *X*^*2*^* *= 0.024, *df* = 1, *P* = 0.877).

### Gift acceptance increases offspring production

**Fig. 4.**
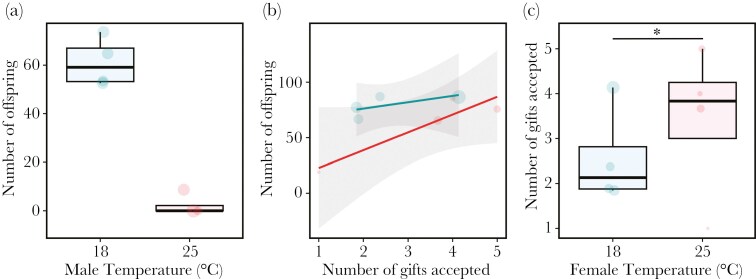
**(a) The effect of male development temperature on the number of offspring produced.** Males exposed to either 18 °C (BF1 *n* = 30, BF14 *n* = 21, BF7 *n* = 19, DG7 *n* = 20) or 25 °C (BF1 *n* = 5, BF14 *n* = 9, BF7 *n* = 10, DG7 *n* = 13) throughout development mated. After copulation females were left to lay eggs and the number of offspring produced were counted. **(b) The effect of female temperature and the number of gifts accepted on offspring production in fertile pairs.** One male and one female from either treatment (18 °C or 25 °C) from the same line were introduced into a chamber and mated females were left in a vial for 30 d to lay eggs. The total number of offspring produced were counted to monitor long-term offspring production. As 25 °C males were almost completely sterile (Fig. 4A) we examined offspring production in females (blue 18 °C or red 25 °C) that accepted a gift from a fertile control male. **(c) The effect of mated female temperature on the number of gifts accepted from fertile control males.** Each point represents the mean value for each isofemale line and the diameter of each point is representative of sample size (see Table S3).

Developmental heat stress induced nearly 100% male sterility (Fig. 4A). Given this result, we next examined the effect of female temperature and the number of gifts accepted on offspring number in fertile pairs with a control male. As we only included control males in the analysis, and females that developed at 25 °C were less likely to mate, the sample size for this analysis is smaller than other analyses (see Table S3). The number of gifts accepted positively correlated with the number of offspring produced (Fig. 4B; *t* = 2.189, *P* < 0.05), and this relationship was consistent across both female temperature treatments (Log-likelihood: *X*^*2*^* *= 0.881, *df* = 1, *P* = 0.348). There was no significant difference in the number of offspring produced between female temperature treatments (ANOVA: *X*^*2*^* *= 2.639, *df* = 1, *P* = 0.104). However, mated heat-stressed females accepted significantly more gifts than control females before mating (Fig. 4C; *Estimate* = 0.559 ± 0.25, *z* = 2.274, *P* < 0.05), which may have allowed them to increase progeny production to the same level as control females.

## Discussion

In this study, we show that developmental heat stress in both sexes strongly influences nuptial gift behavior and reproductive output in *D. subobscura*. Heat-stressed males were less likely to present a gift, and if a gift was presented, gift acceptance probability decreased while latency to accept gifts increased. Additionally, both control and heat-stressed males were less likely to present a gift to heat-stressed females. The number of gifts presented was mediated by an interaction between male and female heat treatment: heat-stressed males presented significantly fewer gifts compared to control males only when paired with heat-stressed females. Heat stress also reduced mating likelihood in both sexes and rendered males largely infertile. Among fertile individuals, gift acceptance increased offspring production in both female treatments, but heat-stressed females accepted more gifts to achieve the same reproductive output as control females. These findings highlight that developmental temperature affects courtship and reproductive output in this species. While both sexes were negatively impacted by increased temperature, sex-specific effects on gift presentation and acceptance suggest that mate choice dynamics may shift in response to warming conditions, impacting sexual selection processes and reproductive outcomes.

Developmental heat stress in both sexes reduced the probability of gift presentation, independent of the opposite sex’s temperature treatment, suggesting that heat stress may impair typical courtship behavior. In males, this reduction in nuptial gift presentation may represent an honest signal of male fertility, as the majority of heat-stressed males were sterile. How developmental temperature changes gift presentation remains unclear. However, as *D. subobscura* nuptial gifts consist of regurgitated crop content (Steele 1986a), heat stress may impair feeding behavior, reducing food intake and crop fullness, inhibiting regurgitation during courtship. Supporting this hypothesis, starved *D. subobscura* males are less likely to produce a gift compared to control males, indicating food consumption influences gift presentation ability (Steele 1986b;Immonen et al. 2009). Alternatively, developmental heat stress may physiologically impair the crop (eg. reduce size) or its neuronal control systems (Tanaka et al. 2017; Sato et al. 2020), negatively impacting food processing, storing and/or regurgitation (Stoffolano Jr and Haselton 2013). In black blow flies (*Phormia regina*), for example, only individuals with sufficiently large crops can regurgitate (Stoffolano Jr et al. 2008).

A recent study on *D. subobscura*, using long term laboratory populations, found male heat stress reduced the probability of gift presentation in a Spanish, but not a Dutch, population (Grandela et al. 2024). Notably, this previous study substantially differed from ours in that only males were heat stressed, males were exposed to 26 °C throughout pupation and adulthood, and nuptial gift behavior was not directly assessed. Given that our study examined gift-giving in a UK population, these findings may collectively suggest that the effect of heat stress on pre-copulatory behavior varies geographically. Population-specific range margin effects (Kawecki 2008), gene swamping (Hoffman and Blows 1994), or chromosomal inversions—which have been shown to vary latitudinally (Krimbas 1993; Rezende et al. 2010) and respond to thermal shifts in the environment (Rodríguez-Trelles et al. 2013; Rodríguez-Trelles and Tarrio 2024)—could contribute to this variation. Additionally, previous work has demonstrated geographical differences in thermal sensitivity to fertility (Porcelli et al. 2017; Santos et al. 2023), sperm motility (Porcelli et al. 2017), and testes gene expression (Porcelli et al. 2016). Such differences may alter the trade-off between pre- and post-copulatory investment following heat stress, driving variation in male gift giving behavior. Indeed, trade-offs between nuptial gifts and other sexually selected traits have been noted across taxa (Jarrige et al. 2013; Barbosa et al. 2015). Overall, the geographical differences in behavioral sensitivity to temperature observed between studies emphasizes the need to investigate temperature effects across multiple populations to better understand species’ responses to climate warming.

Previous research has demonstrated male mate choice via differential gift allocation in gift-giving species (eg Jarrige et al. 2013; Solano-Brenes et al. 2021), with males increasing their investment in response to high quality mates. Strategic distribution of resources is expected to vary depending on male condition, given the energetic cost of gift production, as seen, for example, in scorpion flies (*Panorpa cognata*) under nutritional stress (Engqvist and Sauer 2001). As heat stress reduces energy production (Chown and Nicolson 2004), the energy used to maintain essential physiological processes may come at the expense of reproductive traits (Angilletta et al. 2003; Ratz et al. 2024), increasing the cost of gift giving. Consistent with these ideas, heat-stressed males gave significantly fewer gifts to heat-stressed females, demonstrating greater selectivity than control males who were comparatively indiscriminate. Heat-stressed females required more nutritious gifts to achieve the same reproductive output as control females, aligning with previous findings examining the impact of diet restriction on gift behavior (Immonen et al. 2009). Therefore, heat-stressed males may prioritize investment in control fertile females who will require fewer resources provided by gifts to ensure high reproductive output. As heat-stressed males are more constrained in producing gifts, increased investment towards control females will likely benefit these males more than control males. Multiple cues likely contribute to pre-copulatory mate discrimination by males (Jennions and Petrie 1997). For example, variation in female cuticular hydrocarbons alters female attractiveness in *Drosophila montana* (Jennings et al. 2014), and developmental heat stress influences hydrocarbon profiles in *Drosophila*, affecting mating preferences (Baleba et al. 2024). Additionally, developmental temperature may alter visual courtship cues by the female. For example, diet stress increases spontaneous movement in *D. subosbcura* females (Connolly 1966), impairing the males’ ability to track motion patterns—a key component of courtship (Steele 1986b). If developmental heat stress produces a similar effect, then atypical female movement may act as a male assessment mechanism. However, additional work is needed to investigate these hypotheses.

When a gift was presented, females were less likely and took longer to accept gifts from heat-stressed males, despite the reproductive benefits of gift consumption (here; Steele 1986a, b; Immonen et al. 2009). As gift acceptance typically precedes a copulation attempt by the male, reduced acceptance and increased latency suggests female discrimination against heat-stressed males. In no-choice experiments, mate rejection occurs when the cost of mating outweighs the cost of choosiness, as future mating opportunities are not guaranteed (Dougherty and Shuker 2015). Given heat stress caused near-complete male sterility (here; Porcelli et al. 2017; Grandela et al. 2024), females incur substantial fitness costs when mating with heat-stressed males. In monandrous species, these costs are particularly acute relative to polyandrous systems that buffer against male sterility by remating (Price et al. 2010; Sutter et al. 2019; Vasudeva et al. 2021), intensifying selection for pre-copulatory mate discrimination (Hosken et al. 2009). The mechanisms underlying female mate discrimination remain unclear, although changes to male courtship behavior, gift presentation latency, and/or properties of the nuptial gift (eg size, nutrient content etc.) (Steele 1986b) may signal male fertility. For example, male developmental temperature exposure influenced both gift size and the proportion of body mass invested in gifts in the seed-feeding beetle *Callosobruchus maculatus* (Fox et al. 2006). Developmental heat stress may also disrupt other traits that influence female choice, such as body size (Kingsolver and Huey 2008) and/or cuticular hydrocarbons (Savarit and Ferveur 2002; Etges et al. 2017; Rajpurohit et al. 2021; Baleba et al. 2024). However, although nutritional stress affected body size in *D. subobscura*, body size did not influence male gift production or female acceptance (Immonen et al. 2009). As female pre-copulatory discrimination can minimize the negative effects of heat-induced male sterility, stronger selection on nuptial gifts—such as quantity, size or nutritional content—and/or other male fertility indicator traits, is expected as climate warming increases (Wiens 2001; Martínez Villar et al. 2023). Future studies should therefore explore how developmental temperature influences properties of the nuptial gift, to determine whether they facilitate female mate assessment and, if so, how temperature shifts affect selection on these traits.

Examining the effects of heat stress on pre-copulatory behavior is crucial for predicting species’ responses to climate change, given its important yet often overlooked role in mating outcomes and population fitness (Leith et al. 2021). Here, we focus on nuptial gifts, finding that developmental temperature has sex-specific effects in *D. subobscura*, suggesting that nuptial gifts offer direct benefits to females by improving fecundity while also signaling male fertility. Females were less willing to accept gifts from, and therefore mate with, heat-induced sterile males. While this may benefit population persistence in the short term, reduced female mating probability was also observed which could threaten long-term population stability. Our results also demonstrate the potential for male mate choice in this system, suggesting selection may drive discrimination against heat-stressed females when investing in valuable nuptial gifts. As global temperatures rise, both the cost of male gift giving and the frequency of temperature-induced male sterility are likely to increase, driving greater mate selectivity and resource allocation to mitigate heightened fitness costs in both sexes. These changes may alter co-evolutionary dynamics and intensify sexual conflict over reproductive investment, particularly in this monandrous system, where high male investment may limit future mating opportunities, and females risk exclusively pairing with an infertile male. While our focus has been on developmental heat stress, elevated temperature exposure during adulthood may also interfere with nuptial gift behavior. Although untested, both male courtship effort (Conrad et al. 2017; Macchiano et al. 2019; Rosenthal and Elias 2019, 2025; Ratz et al. 2024) and female mate discrimination (Miwa et al. 2018; Brandt et al. 2020) are known to change under adult heat stress in ectotherms. Overall, future studies examining the impact of heat stress across multiple pre-copulatory traits and life stages will contribute to understanding the broader evolutionary and ecological consequences of a warming world.

## Supplementary Material

araf049_suppl_Supplementary_Materials

## Data Availability

Analyses reported in this article can be reproduced using the data provided by Pembury Smith et al. (2025).
