# Supplementary material for: Elevated temperatures have sex-specific effects on nuptial gift behavior
Source: Behav Ecol. 2025 May 8;36(4):araf049. doi: 10.1093/beheco/araf049 (PMC12125707; doi:10.1093/beheco/araf049)
Supplement: araf049_suppl_Supplementary_Materials [file araf049_suppl_supplementary_materials.docx]

**Supplementary data for**

**Elevated temperatures have sex-specific effects on nuptial gift behavior**

Matilda Q.R. Pembury Smith^1, 2^*, Laura Latkova^1^ and Rhonda R. Snook^1, 2^.

***Corresponding author:** Department of Zoology, Stockholm University, Stockholm, SE-106 91, Sweden. Email: matilda.pembury.smith@zoologi.su.se

**Affiliations:**

^1^Department of Zoology, Stockholm University, Stockholm, SE-106 91, Sweden.

^2^Bolin Centre for Climate Research, Stockholm University, Stockholm, SE-106 91, Sweden.

| **Table S1. Description of the statistical models used for each trait analyzed.** Sample size indicates the total number of individuals analyzed (see Table S3 for individual line sample sizes). Transform specifies whether and how the response variable was transformed. Family denotes the model family used. *X^2^* and *p* represent the model statistics for included covariates or line effects. Female Temp and Male Temp are the developmental temperatures of each sex (18°C or 25°C). Time refers to the duration of behavioural observations. Line represents the four isofemale lines used. Gift Giving Latency refers to the duration between pair introduction and the male’s presentation of a nuptial gift. No. Gifts Given and No. Gifts Accepted is the total number of gifts presented/accepted over the observation period. | | | | | | | |
| --- | --- | --- | --- | --- | --- | --- | --- |
| **Response variable** | **Model** | **Sample size** | **Transform** | **Family** | **Covariate/Line** | ***X^2^*** | ***p*** |
| Gift given | Female Temp*Male Temp + Line | 335 |  | Binomial | Line | 8.182 | < 0.05 |
| Gift giving latency | Female Temp*Male Temp + Line | 249 | Log | Gaussian | Line | 10.055 | < 0.05 |
| Number of gifts given | Female Temp*Male Temp + Time + Line | 249 |  | Negative binomial | Time | 33.285 | < 0.001 |
|  |  |  |  |  | Line | 15.519 | < 0.01 |
| Gift acceptance | Female Temp*Male Temp + Line | 229 |  | Binomial | Line | 0.152 | 0.985 |
| Gift acceptance latency | Female Temp*Male Temp + Gift Giving Latency + Line | 187 | Log | Gaussian | Log(Gift Giving Latency) | 229.215 | < 0.001 |
|  |  |  |  |  | Line | 3.331 | 0.343 |
| Number of gifts accepted | Female Temp*Male Temp + No. Gifts Given + Time + Line | 187 |  | Negative binomial | No. Gifts Given | 31.837 | < 0.001 |
|  |  |  |  |  | Time | 16.469 | < 0.001 |
|  |  |  |  |  | Line | 9.063 | <0.05 |
| Mating occurrence | Female Temp*Male Temp + Line | 187 |  | Binomial | Line | 0.326 | 0.568 |
| Mating latency | Female Temp*Male Temp + Line | 123 | Log | Gaussian | Line | 1.612 | 0.657 |
| Mating duration | Female Temp*Male Temp + Line | 123 | Log | Gaussian | Line | 14.652 | < 0.01 |
| Offspring production | Female Temp + No. Gifts Accepted + Line | 65 |  | Gaussian | Line | 2.410 | 0.492 |
| Number of gifts accepted (mated individuals) | Female Temp + Line | 65 |  | Negative binomial | Line | 10.305 | < 0.05 |

| **Table S2. Model selection for offspring production analysis using log-likelihood.** Linear model comparison was conducted to assess the effects of female temperature, the number of gifts accepted, and their interaction using the *anova()* function. Akaike Information Criterion (AIC) measures model quality given the data set by balancing goodness of fit and model complexity. Log-likelihood (LogLik) assesses how well the model explains the observed data given model parameters. Deviance represents how well each model fits the data by quantifying the discrepancy between the observed data and the model’s predictions. *X^2^* is the test statistic for model comparison. Degrees of freedom (*df*) represents the difference in the number of parameters between models. P-value (*p*) indicates whether the difference in model fit is statistically significant. The final model used is in bold. | | | | | | |
| --- | --- | --- | --- | --- | --- | --- |
| **Model** | **AIC** | **LogLik** | **Deviance** | ***X^2^*** | ***df*** | ***p*** |
| **Female Temperature + Number of gifts accepted** | **614.97** | **-300.49** | **600.97** |  |  |  |
| Female Temperature*Number of gifts accepted | 616.09 | -300.04 | 600.09 | 0.881 | 1 | 0.348 |

| **Table S3. The total and individual line sample sizes for each mating combination for each trait analyzed.** Row header describes the mating combination (e.g., 18M18F is a control male paired with a control female). No pairs including a male that developed at 25°C were included in the offspring production analysis. | | | | | | | | | | | | | | | | | | | | | | |
| --- | --- | --- | --- | --- | --- | --- | --- | --- | --- | --- | --- | --- | --- | --- | --- | --- | --- | --- | --- | --- | --- | --- |
| **Variable** | **18M18F** | | | | | **18M25F** | | | | | **25M18F** | | | | | | **25M25F** | | | | | |
|  | **BF1** | **BF14** | **BF7** | **DG7** | **Total** | **BF1** | **BF14** | **BF7** | **DG7** | **Total** | **BF1** | **BF14** | **BF7** | **DG7** | **Total** | **BF1** | | **BF14** | **BF7** | **DG7** | **Total** |  |
| Gift presentation probability | 35 | 26 | 23 | 20 | 104 | 13 | 19 | 24 | 20 | 76 | 9 | 15 | 31 | 23 | 78 | 7 | | 15 | 29 | 26 | 77 |  |
| Gift presentation latency  The number of gifts presented | 35 | 22 | 18 | 18 | 93 | 11 | 13 | 16 | 14 | 54 | 7 | 11 | 19 | 17 | 54 | 6 | | 7 | 19 | 16 | 48 |  |
| Gift acceptance probability | 29 | 21 | 16 | 17 | 83 | 10 | 12 | 16 | 13 | 51 | 7 | 10 | 17 | 17 | 51 | 6 | | 5 | 19 | 14 | 44 |  |
| Gift acceptance latency  The number of gifts accepted  The probability of mating | 29 | 20 | 16 | 14 | 79 | 8 | 10 | 15 | 13 | 45 | 3 | 8 | 10 | 15 | 36 | 5 | | 3 | 12 | 6 | 26 |  |
| Mating latency  Mating duration | 25 | 14 | 13 | 11 | 63 | 2 | 7 | 9 | 10 | 28 | 3 | 5 | 3 | 10 | 21 | 2 | | 1 | 5 | 3 | 11 |  |
| Offspring production & number of gifts accepted (female mated to a fertile 18°C male)  *female temperature effect* | 22 | 13 | 8 | 9 | 52 | 1 | 2 | 4 | 6 | 13 | NA | NA | NA | NA | NA | NA | | NA | NA | NA | NA |  |

| **Table S4. Mean and standard error for each mating combination for each trait analyzed.** Row header describes the mating combination (e.g., 18M18F is a control male paired with a control female). No pairs involving 25°C males were included in the offspring production analysis as these males are sterile. | | | | | | | | | | | | | | |
| --- | --- | --- | --- | --- | --- | --- | --- | --- | --- | --- | --- | --- | --- | --- |
| **Variable** |  | **18M18F** | | | **18M25F** | | | **25M18F** | | | **25M25F** | | | |
|  |  | **Mean** | **SE** | **n** | **Mean** | **SE** | **n** | **Mean** | **SE** | **n** | **Mean** | **SE** | **n** |  |
| Gift presentation | Yes |  |  | 93 |  |  | 54 |  |  | 54 |  |  | 48 |  |
|  | No |  |  | 11 |  |  | 22 |  |  | 24 |  |  | 29 |  |
| Gift presentation latency |  | 18.2 | 2.0 | 93 | 26.3 | 3.5 | 54 | 25.4 | 3.2 | 54 | 26.5 | 3.2 | 48 |  |
| The number of gifts presented |  | 10.8 | 1.3 | 93 | 11.4 | 1.6 | 54 | 14.6 | 2.0 | 54 | 9.0 | 1.6 | 48 |  |
| Gift acceptance | Yes |  |  | 79 |  |  | 46 |  |  | 36 |  |  | 26 |  |
|  | No |  |  | 4 |  |  | 5 |  |  | 14 |  |  | 17 |  |
| Gift acceptance latency |  | 23.7 | 2.3 | 79 | 32.9 | 3.9 | 46 | 35.7 | 4.5 | 36 | 40.3 | 3.9 | 26 |  |
| The number of gifts accepted |  | 3.1 | 0.3 | 79 | 3.7 | 0.5 | 46 | 3.2 | 0.4 | 36 | 2.5 | 0.4 | 26 |  |
| The probability of mating | Yes |  |  | 63 |  |  | 28 |  |  | 21 |  |  | 11 |  |
|  | No |  |  | 16 |  |  | 18 |  |  | 15 |  |  | 15 |  |
| Mating latency |  | 36.6 | 3.2 | 63 | 47.3 | 6.6 | 28 | 49.5 | 7.5 | 21 | 46.1 | 8.3 | 11 |  |
| Mating duration |  | 12.9 | 1.0 | 63 | 11.8 | 1.1 | 28 | 16.3 | 1.9 | 21 | 16.2 | 3.2 | 11 |  |
| Offspring production |  | 80.9 | 3.6 | 52 | 68.2 | 7.7 | 13 | NA | NA | NA | NA | NA | NA |  |
| The number of gifts accepted |  | 2.9 | 0.3 | 52 | 3.9 | 1.0 | 13 | NA | NA | NA | NA | NA | NA |  |
